# Carcinogenic Medications: A Review of Specific Agents and Molecular Mechanisms of Carcinogenesis

**DOI:** 10.1002/cnr2.70538

**Published:** 2026-04-07

**Authors:** Desta Seyoum Tadesse, Kalkidan Tekletsadik, Berhan Begashaw, Awgichew Shewasinad Yehualashet, Awol Mekonnen Ali, Kassahun Dires Ayenew

**Affiliations:** ^1^ Department of Pharmacology, Asrat Woldeyes Health Science Campus Debre Berhan University Debre Berhan Ethiopia

**Keywords:** carcinogenesis, chemotherapy, drug‐induced cancer, hormone therapy, immunosuppressants, medications, molecular mechanisms, pharmacovigilance

## Abstract

**Background:**

Pharmacovigilance has revealed an alarming correlation between certain medications and a higher risk of cancer. In this narrative review, included research from 2020 to 2025, along with a few seminal older studies, so that it provides a clear picture of which drugs actually set off cancer and mechanisms involved at the molecular level. An extensive literature review of the subject was designed on PubMed, Embase, Scopus, and Web of Science using systematic search. Search words and phrases included: “Carcinogenic drugs,” “drug‐induced cancer,” “medication‐induced carcinogenesis,” “immunosuppressant cancer risk,” “hormone therapy and cancer,” “chemotherapy‐induced secondary malignancies,” and names of relevant drugs.

**Recent Findings:**

Our discussion covers a broad spectrum of drugs, including immunosuppressants, hormone therapies, and chemotherapy agents that can unsurprisingly cause secondary cancers. The review has addressed that there are other medications with solid evidence linking them to cancer. The review focuses on how cancers are induced by chemicals through DNA damage, epigenetic modifications, chronic inflammation, immunological suppression, and receptor‐mediated signaling pathways.

**Conclusion:**

A thorough understanding of the risk and mechanisms is essential for the design of safer therapies, implementation of risk reduction strategies, and advancement of informed prescribing practice.

## 1 Introduction

Drug therapy is the backbone of modern medicine to treat acute conditions and chronic diseases, often prolonging patients' lives in addition to improving quality of life. However, there are inherent risks resulting from pharmacotherapy [1]. Undoubtedly, the drug‐related adverse reaction is one of the major adverse effects that are usually underestimated—drug‐induced carcinogenesis, that is, the ability of a drug to cause or promote the development of a cancer [2]. The aim of drug therapy is to cure or ameliorate any condition, but several drugs nowadays have been identified to be causal agents in cancer rather than being curative agents [2, 3].

Carcinogenicity is a critical and complicated condition and results from many factors over time. Some of the risks are intrinsic to the person, but quite a number are imparted from the internal and external sources, referred to as carcinogenic risk modifiers—it can be increased or decreased depending on the person's susceptibility to cancer. These modifiers are important in terms of prevention and individual risk assessment, with examples such as lifestyle, exposure to the environment, genetics, and even the combination of medicines [3, 4].

Drug induced carcinogenesis typically involves different pathways. Some drugs exhibit a true carcinogenic and mutagenic effect that directly damage DNA [4, 5]. Others cause inhibition of the immune system from working properly [6, 7]. With this knowledge, physicians should take a keen interest in knowing which kinds of medicines potentially have carcinogenic risk and how they select and prescribe them because of the general use of these drugs [1, 2, 8]. Chemotherapy is appropriate and mostly considered as a last option because of its carcinogenic effect, other toxicities, and many malignancies are caused by substances that have long been thought to be beneficial [7]. By doing so, they are better able to weigh risks versus benefits, optimize prescribing practices, and keep high‐risk patients on preventive treatment [5, 9].

An explanation of the molecular mechanism, the pattern of occurrence in the population, and the practicality in the clinical setup are all included in this comprehensive assessment of the literature on the association between the aforementioned medicines and cancer. With this kind of portrayal, we hope to alert medical practitioners in a way that facilitates the process of administering the appropriate medication while also drawing attention to the areas that require more study.

## 2 Methods

An extensive literature review of the subject was designed on PubMed, Embase, Scopus, and Web of Science using systematic search. Search words and phrases included: “Carcinogenic drugs,” “drug‐induced cancer,” “medication‐induced carcinogenesis,” “immunosuppressant cancer risk,” “hormone therapy and cancer,” “chemotherapy‐induced secondary malignancies,” and names of relevant drugs, for example, “azathioprine,” “tamoxifen” altered with “carcinogenesis” or “cancer risk.” This search was limited to articles published during the past decade (2020–2025), with the exception of landmarks in the field critical for background. Both review articles and original works were included. Seminal and foundational publications from before 2020 were included due to their pertinence to the review.

The review addressed published original research articles, systematic reviews, meta‐analyses, and rigorous case reports/series with very high levels of evidence of causality. To achieve a balanced review and critical evaluation about agents with disputed carcinogenic potential such as mycophenolate mofetil, we made a deliberate effort to identify and include studies with conflicting results.

### 2.1 Eligibility/Inclusion Criteria

- Reports of carcinogenicity by drugs in either humans or animal models.
- Articles that elucidate the molecular processes by which drugs cause cancer.
- Systematic reviews and meta‐analyses.
- Articles of case reports and case series with very convincing evidence for drug‐induced carcinogenesis.

### 2.2 Exclusion Criteria

- Studies of cancer not including a second malignancy from the chemotherapeutic agent.
- Studies without the English language.
- Research whose relevance to human health is minimal (such as purely environmental toxicology research).
- Research without clear evidence of a causal relationship between drug exposure and cancer induction.

### 2.3 Data Extraction and Synthesis

Extraction includes the drug name, cancer associated, study design, population, molecular pathways, and significant risk factors. A narrative synthesis was adopted to summarize key themes and knowledge gaps. A total of 250 revisions were analyzed, and 187 were included for final review.

## 3 Medications With Carcinogenic Potential

### 3.1 Immunosuppressants

In addition to treating autoimmune diseases, medical practitioners administer immunosuppressive drugs to transplant recipients in order to prevent organ rejection [10]. However, the pharmaceutical treatments change the likelihood of developing some cancers, such as cutaneous tumors and lymphomas (Table 1) [24].

- Purine analogues (e.g., azathioprine): Purine analogue azathioprine is a commonly used immunosuppressive medication [11]. Studies have linked its use to an increased incidence of squamous cell carcinoma and other non‐melanoma skin malignancies (NMSCs) [12]. It is thought to work by increasing the integration of azathioprine's metabolites into the DNA, which damages the DNA and causes mutations [13]. Furthermore, azathioprine may make it more difficult for the immune system to identify and eliminate cancer cells [11].
- Calcineurin inhibitors (e.g., cyclosporine and tacrolimus): Additionally, these calcineurin inhibitors raise the incidence of lymphomas and skin malignancies [14]. The immune system's capacity to recognize cancerous cells is weakened by cyclosporine and tacrolimus, which disrupt T‐cell activity [15]. Additionally, they have the ability to promote the development of new blood vessels, which aids in tumor growth and metastasis [16].
- IMPDH inhibitors (e.g., mycophenolate mofetil (MMF)): MMF inhibits purine production, thus reducing lymphocyte proliferation [17]. Its carcinogenicity is considered by some to be less definite than calcineurin inhibitors (CNIs) such as cyclosporine and azathioprine; however, its associated carcinogenicity remains controversial. Some studies indicate that MMF may increase the risk of skin cancers and lymphomas; however, other clinical data and long‐term cohort studies have not consistently demonstrated a strong link between MMF and cancer risk compared with other potent immunosuppressants [18, 19]. This appears to have created contradictions in the literature, which warrants careful consideration in evaluating its risk profile.
  There are also multiple confounding factors that elaborate diverse effects of MMF. Most of the time, MMF is not used alone but is included in a regimen with other potent immunosuppressant drugs, thus making it hard to attribute the cancer risk to MMF alone. The prevailing trend currently is to agree that MMF is less carcinogenic than azathioprine and calcineurin inhibitors. Its risk is likely dependent on the intensity and duration of the whole immunosuppressive burden, past oncogenic viral exposures, and individual susceptibility. This makes it a good agent in schemes directed to reducing carcinogenesis associated with immunosuppression [25, 26, 27].
- Biological immunosuppressants (TNF‐alpha inhibitors, etc.): Researchers have linked anti‐TNF medications to an increased risk of skin cancer and lymphoma. Patients with rheumatoid arthritis and other autoimmune diseases can attest to this [20, 21].
- mTOR inhibitors (sirolimus, everolimus) paradoxically: Though mTOR inhibitors are generally considered anti‐oncogenic and are used for the treatment of certain cancers, inhibition of various pathways essential for the growth and proliferation of cells (i.e., PI3K/AKT/mTOR pathway) does not completely avert these cancers due to the immunosuppressive nature of the drugs [22]. They exhibit a dualistic effect, meaning they exhibit opposing effects. In already existing cancers, mTOR inhibitors exert anti‐proliferative, and anti‐angiogenic effects. However, in the case of de novo carcinogenesis: Because of the high level of immunosuppression, this seems to outweigh against such direct anti‐cancer effects. mTOR inhibitor net oncologic risk profile is favorable compared to calcineurin inhibitor, specifically virus‐associated malignancies. While the use of an mTOR inhibitor certainly brings with it the risk of adverse effects, it is considered to be safer than CNI in terms of effects on the nephron. This is because it does not activate direct pro‐oncogenic pathways (upregulation of TGF‐β, induction of VEGF) that are associate with CNIs. Therefore, while comparing regimens, the trade‐off of immunosuppression with mTOR has often been preferred against combined immunosuppression and direct pharmacological cancer promotion seen with CNI [23].

**TABLE 1:** Immunosuppressants and their associated cancer risks.

Drug class	Example drugs	Associated cancers	Proposed mechanisms	References
Purine analogues	Azathioprine, 6Mercaptopurine	Squamous cell carcinoma; Non‐melanoma skin cancer (NMSC); Post‐transplant lymphoproliferative disorder (PTLD)	Metabolites incorporate into DNA → DNA damage and mutations; UVA photosensitization → reactive oxygen species → p53 mutations; Impaired immune surveillance	[11, 12, 13]
Calcineurin inhibitors	Cyclosporine, Tacrolimus	Non‐melanoma skin cancer (NMSC); Post‐transplant lymphoproliferative disorder (PTLD); Kaposi sarcoma	T‐cell suppression → impaired cancer cell recognition; TGF‐β upregulation → epithelial‐mesenchymal transition; VEGF induction → angiogenesis; Inhibition of DNA repair	[14, 15, 16]
IMPDH inhibitors	Mycophenolate mofetil (MMF)	Non‐melanoma skin cancer (potential risk); Post‐transplant lymphoproliferative disorder (potential risk)	Inhibition of purine synthesis → reduced lymphocyte proliferation; No direct pro‐angiogenic effects; Risk confounded by concomitant immunosuppressive therapy; Lower carcinogenic potential compared to azathioprine and CNIs	[17, 18, 19]
Biologic agents	TNF‐α inhibitors (Infliximab, Adalimumab, Etanercept)	Non‐melanoma skin cancer; Lymphoma	Immune modulation → impaired anti‐tumor immunity; Specific risk profiles vary by agent and underlying disease	[20, 21]
mTOR inhibitors	Sirolimus, Everolimus	Lower risk compared to CNIs for most malignancies; Continued risk for virus‐associated cancers	Dual effect: immunosuppression + direct anti‐proliferative effects; No upregulation of TGF‐β or VEGF; Anti‐angiogenic properties may offset carcinogenic risk	[22, 23]

### 3.2 Hormonal Therapies

Hormone therapies, e.g., estrogen and androgenic medications, are carcinogenic in hormone‐responsive tissues (Table 2) [2, 35].

- Estrogen therapy: Unopposed estrogen therapy—the administration of estrogen, particularly when progesterone is not present—has been linked to an increased risk of endometrial cancer development [9]. Estrogen directly promotes the proliferation of endometrial cells, as demonstrated in experimental models using endometrial cancer cell lines, which raises the risk of cellular mutation and the development of cancer [28]. By counteracting the proliferative impact of estrogen, progestin can somewhat lower the risk when used in treatment [29, 30].
- Selective estrogen receptor modulators (SERMs): It has been demonstrated that tamoxifen, a SERM used to treat breast cancer, raises the risk of endometrial cancer, but less so than unopposed estrogen [31]. Tamoxifen raises the risk of cancer by increasing cell proliferation in the endometrium, where it acts as an estrogen agonist [32].
- Androgen deprivation therapy (ADT): ADT, which is used to treat prostate cancer, has also been linked to an increased risk of metabolic syndrome and cardiovascular disease [33, 34]. ADT can cause hormonal imbalances and metabolic changes that may encourage the development of secondary malignancies, even though it is not carcinogenic to the prostate itself [34].

**TABLE 2:** Hormonal therapies and their associated cancer risks.

Drug class	Example drugs	Associated cancers	Clinical context	Proposed mechanisms	Evidence level	Citations
Estrogen therapy (unopposed)	Conjugated estrogens, Estradiol	Endometrial cancer	Menopausal hormone therapy	Direct endometrial proliferation; genotoxic metabolite generation; chromosomal instability from mitotic errors	Strong	[9, 28]
Estrogen + progestin combined therapy	Combined HT	Breast cancer (risk varies by regimen)	Menopausal hormone therapy	Progestin‐mediated proliferation; complex receptor interactions	Moderate	[29, 30]
SERMs	Tamoxifen, Raloxifene	Endometrial cancer	Breast cancer treatment and prevention	Estrogen agonist in endometrium; cell proliferation; DNA damage from metabolites	Strong	[31, 32]
Androgen deprivation therapy (ADT)	GnRH agonists (Leuprolide, Goserelin), GnRH antagonists (Degarelix)	Potential secondary malignancies (under investigation)	Prostate cancer treatment	Hormonal imbalances; metabolic changes; long‐term effects under investigation	Emerging	[33, 34]

### 3.3 Chemotherapy Agents

Ironically, even though chemotherapy medications are designed to kill cancer cells, a few of them are also able to induce secondary malignancies [36]. Secondary cancers are generally treatment‐related and can arise years after the initial chemotherapy treatment [37].

- Alkylating agents: Alkylating drugs, (e.g., Cyclophosphamide, melphalan, chlorambucil, busulfan), exert cytotoxicity through transfer of alkyl groups to DNA bases, forming DNA crosslinks and adducts. Although the malignant cells are damaged by this mechanism, it can also cause mutagenic damage to normal tissues, especially in the hematopoietic stem cell. In case these lesions happen in critical genes (such as tumor suppressor genes like TP53) and these lesions were not fixed, they can trigger carcinogenesis. This cytotoxic pathway explains the risk of treatment‐associated acute myeloid leukemia (t‐AML) and myelodysplastic syndrome (t‐MDS) that increases according to dose following exposure to alkylating agents. The risk accumulates in terms of dose and length of treatment with a latency of 5–7 years being average [38, 39].
- Topoisomerase II inhibitors: Topoisomerase II inhibitors (e.g., etoposide, teniposide, doxorubicin, daunorubicin) are linked to secondary acute myeloid leukemias that are accompanied by balanced chromosome translocation, most commonly a KMT2A (MLL) gene at chromosome 11q23 [40]. These agents stabilize the topoisomerase II‐DNA complex which results in DNA breaks of the two strands and rearrangement of the chromosomal structure in the process of DNA replication. Topoisomerase inhibitor‐related AMLs have a short latency of 1–3 years of exposure, as compared to the alkylator‐related leukemias (usually proceeding 5–7 years), and are in most instances linked to a small set of oncogenes that are fused into each other [41].
- Platinum‐based agents: Platinum agents (cisplatin, carboplatin, oxaliplatin) induce intra‐ and inter‐strand crosslinks of the DNA resulting in apoptosis in cancerous cells with the potential to cause genomic instability to taxa [42]. Patients who are long term survivors of testicular cancer on platinum drugs have been noted to have extremely high risks of secondary solid tumors (e.g., gastrointestinal, genitourinary, lung) and leukemia [42, 43]. The pathophysiology includes irreversible damage to the DNA, the dysfunction of nucleotide excision repair, and mutations that are accumulated with time, and the risk is still high decades after treatment [44].

### 3.4 Other Medications

**FIGURE 1:**
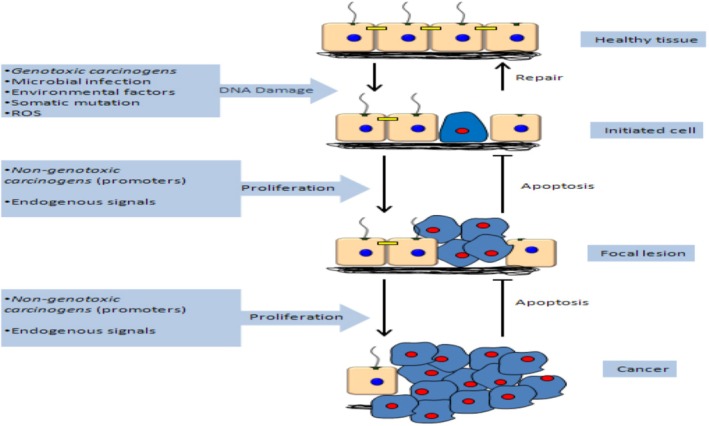
Mechanisms of phenacetin induced renal carcinoma. This schematic describes the pathway of phenacetin induced renal carcinoma that goes through four important phases: Normal Cellular Homeostasis and Architecture Healthy Tissues: Healthy cellular homeostasis and architecture. Initiated Cell: DNA damages take place through exposure to genotoxic carcinogens (e.g., phenacetin metabolites) or because of environmental circumstances. When repair mechanisms do not occur, a mutation is fixed forming an initiated cell. Focal Lesion: It involves selective clonal expansion (proliferation) of the initiated cells through the action of focal death and survival signals to promote carcinogen‐induced neoplasm formation: Non‐genotoxic carcinogens (promoters) and endogenous signals. Cancer: The additional growth of cancer and the lack of apoptosis results in the formation of malignant tumour. Originally created by the authors using BioRender.com (2025).

- Phenacetin: This cancer‐causing substance was once an analgesic and was found in over‐the‐counter pain medications [45]. It was linked to an increased risk of bladder and renal pelvic cancer [46]. Acetaminophen and other chemicals that harm DNA are produced from phenacetin (Figure 1) [47].

- Arsenic trioxide: Arsenic trioxide, which is demonstrated to cure acute promyelocytic leukemia (APL), may cause QT elongation and therefore cardiac arrhythmia [48].

**FIGURE 2:**
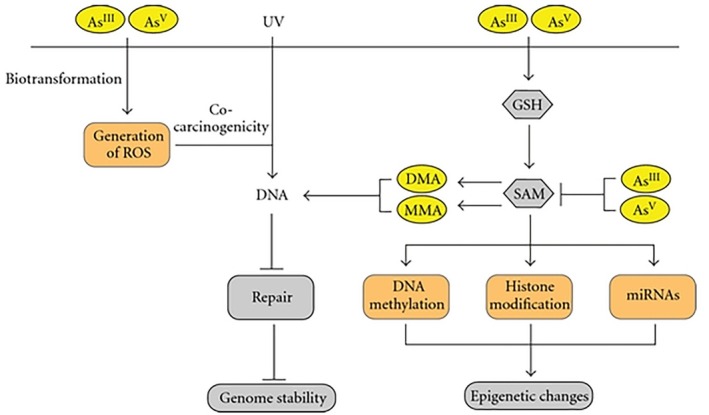
Mechanisms of arsenic‐induced carcinogenesis. Schematic representation of proposed arsenic‐induced carcinogenic mechanisms. Arsenic can enter cells in both tri‐ or pentavalent forms (AsIII or AsV). Inside cells, AsV is converted to AsIII, with subsequent methylation to monomethylated (MMA) and dimethylated (DMA) species. The methylation of inorganic arsenic consumes both S‐adenosylmethionine (SAM) and glutathione (GSH). Cellular damage derived from arsenic biotransformation can occur through generation of reactive oxygen species (ROS), and through epigenetic mechanisms: changes in DNA methylation patterns (by depletion of cellular pools of methyl group), histone modification, and altered expression of microRNAs (miRNAs). Adapted with permission of [49] Martinez et al. (2011), Molecular Cancer, BioMed Central. We have confirmed that this number is under Creative Commons Attribution License which allows modification with proper reference.

As indicated in Figure 2, carcinogenic effects induced by arsenic exposure are mostly generated due to its biotransformation process, having effects at genetic and epigenetic levels. Arsenic biotransformation occurs through a series of cycles of reduction, oxidation, and methylation reactions. Pentavalent arsenic (AsV) is reduced to arsenite (AsIII), using glutathione (GSH) and thioredoxin (TRX) as electron donors. In the excretion process, AsIII is methylated using S‐Adenosyl methionine (SAM) as a source of methyl groups resulting in generation of arsenic species with higher carcinogenic potential. Genetic alterations are largely due to the generation of reactive oxygen and/or nitrogen species, partially derived from arsenic‐induced mitochondrial dysfunction. Epigenetic effects, such as changes in DNA methylation patterns, have been linked to deprivation of SAM. Image adopted from [50].

- Growth hormones: Research shows that growth hormones have the ability to provoke cancers such as colon, prostate, and breast due to their participation in the process of cell proliferation and angiogenesis [39].

### 3.5 Epidemiological Burden and Mechanistic Strength of Carcinogenic Agents

#### 3.5.1 Immunosuppressants

- Epidemiologic burden: The malignance risk conferred by these drugs collectively is 2–3 folds, the most nuisance being through non‐melanoma skin cancer or cutaneous malignancy and post‐transplant lymphoproliferative disorder (PTLD). The risk of these malignancies develops in a multiplicative fashion with increasing intensity of the drug and duration of therapy [51].
- Mechanistic strength: Carcinogenicity is indirect via loss of immune surveillance towards oncogenic viruses (EBV, HPV) and direct through pharmacological activation of oncogenic pathways (e.g., UVA‐mediated DNA damage by AZA; angiogenesis and metastasis driven by TGF‐β and VEGF by CNIs) [52].

#### 3.5.2 Azathioprine

- Epidemiologic burden: Most strongly associated with SCC of the skin, which is often aggressive and ensures rapid onset, particularly in sun‐exposed areas [53].
- Mechanistic strength: Its power is unique; it acts as a direct UVA photosensitizer, generating mutagenic reactive oxygen species that induce those signature p53 mutations, totally independent of its immunosuppressive effect [54].

#### 3.5.3 Calcineurin Inhibitors (Cyclosporine, Tacrolimus)

- Epidemiologic burden: Associated with a whole range of malignancies (NMSC, PTLD, Kaposi's sarcoma) and is a primary driver of post‐transplant cancer risk [55, 56].
- Mechanistic strength: Besides immunosuppression, they exert real pro‐oncogenic effects: TGF‐β‐mediated epithelial‐to‐mesenchymal transition (EMT), VEGF‐induced angiogenesis, and inhibition of DNA repair [57].

#### 3.5.4 Mycophenolate Mofetil (MMF)

- Epidemiologic burden: Epidemiological data are confounded by combination therapy; however, it is shown consistently to bear less carcinogenicity than that associated with the usage of AZA or CNIs [56, 58].
- Mechanistic strength: Lacking direct photocarcinogenic and angiogenic pathways, its risk is largely indirect through antiviral immunosuppression, making it the agent of choice for CNI‐minimization strategies in order to reduce net carcinogenic burden [59].

#### 3.5.5 mTOR Inhibitors (Sirolimus, Everolimus)

- Epidemiologic burden: Carrying favorable net oncologic attributes as compared to CNIs, lower risk for virus‐associated malignancies and NMSC [60].
- Mechanistic strength: They are uniquely paradoxical: their potent anti‐angiogenic and anti‐proliferative effects directly counter carcinogenesis, even though their immunosuppressive effect can allow for viral‐mediated cancers. They lack the direct TGF‐β/VEGF induction seen with CNIs [61].

#### 3.5.6 Hormonal Therapies (e.g., Estrogen, SERMs)

- Epidemiologic burden: The risk is increased in hormone‐responsive tissues (endometrium, breast). The burden is modified by progestin co‐administration and treatment duration [30].
- Mechanistic strength: Receptor‐mediated proliferative signaling, genotoxic estrogen metabolite generation causing DNA damage, and induction of chromosomal instability through mitotic errors drive carcinogenesis [62].

#### 3.5.7 Chemotherapy Agents (Alkylators, Topo‐II Inhibitors)

- Epidemiologic burden: Acute myeloid leukemia (AML) type‐2, an association with these agents causing therapy‐related myeloid neoplasms (t‐MN/AML), exhibits a characteristic latent clinical period with poor prognosis [63].
- Mechanistic strength: Their mechanism is directly genotoxic: causing DNA cross‐linking (alkylators) or DNA double‐strand breaks via topoisomerase II poisoning, leading to chromosomal translocations (e.g., MLL gene rearrangements) that initiate leukemia [64].

## 4 Molecular Mechanisms of Drug‐Induced Carcinogenesis

**FIGURE 3:**
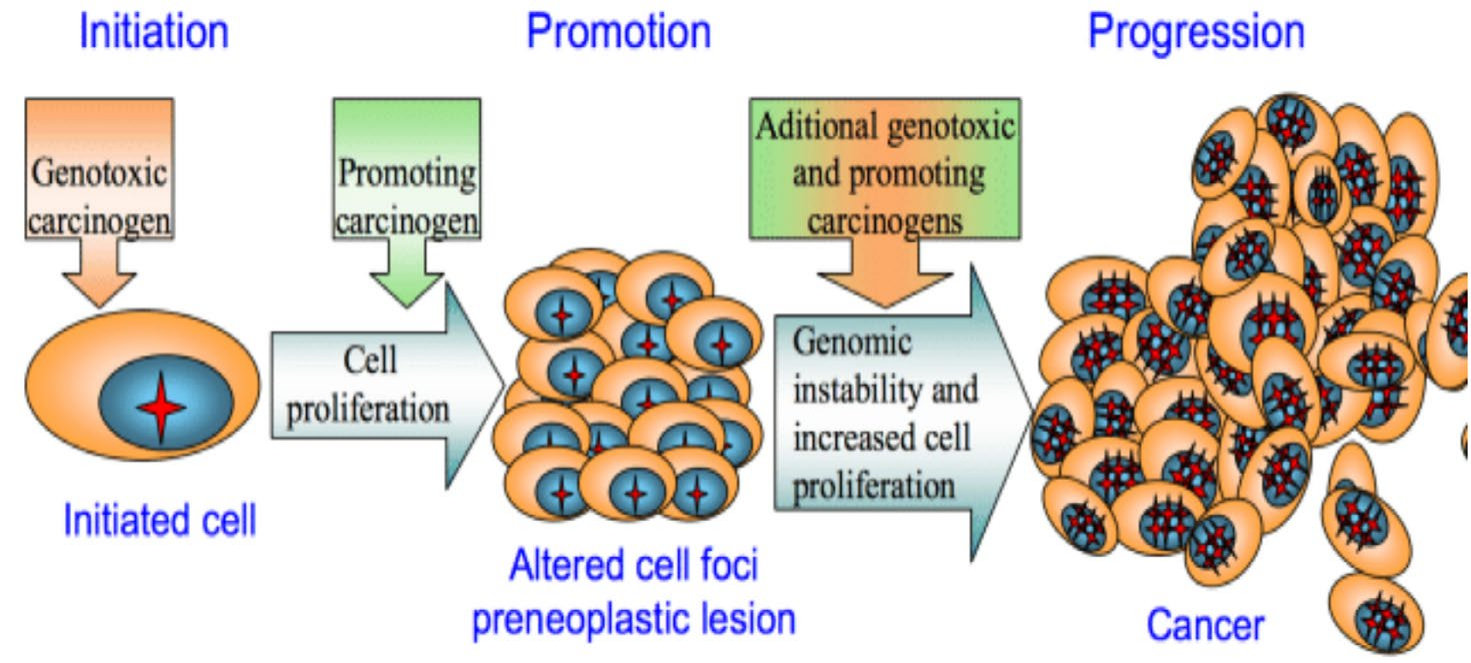
Chemical carcinogenesis mechanisms: Initiation, promotion, and progression. Mechanism of chemical carcinogenesis involves three main stages of carcinogenesis; initiation, promotion and progression. The genotoxic carcinogen produces initiated cells with a mutant genotype, during promotion these cells can be stimulated to proliferate by tumor promoting carcinogens to form clusters of initiated cells, the formed lesion is predisposed to progress into a cancer, but, additional exposition to genotoxic and tumor promoting substances accelerates progression stage by increasing genomic instability and cell proliferation rate to convert a preneoplastic lesion into cancer [94]. Adapted from Basu (2018), International Journal of Molecular Sciences, published under CC BY 4.0 license.

**FIGURE 4:**
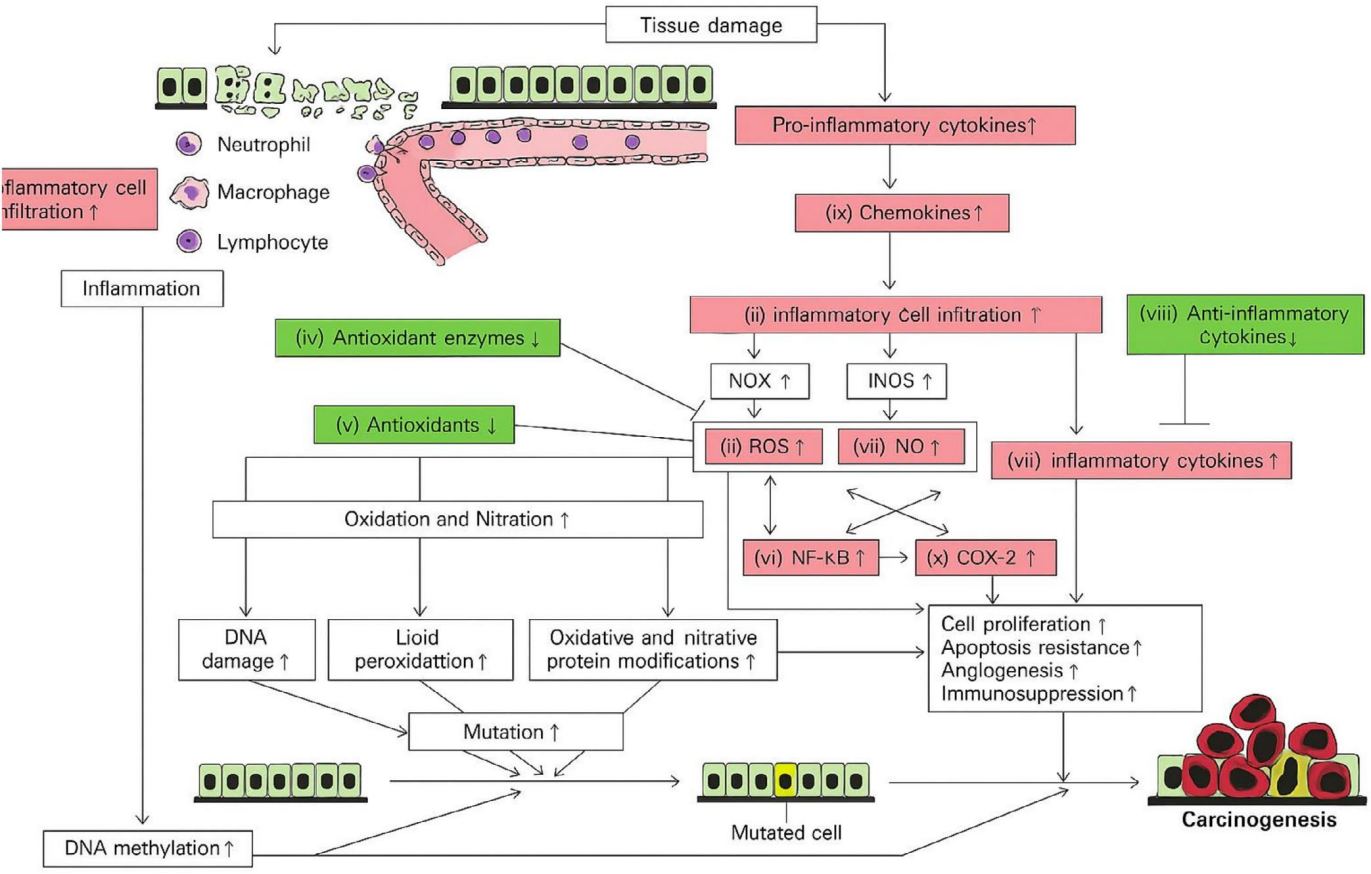
Inflammation‐mediated carcinogenesis pathways. Inflammation‐mediated carcinogenesis results from tissue injury involving different pathways, which include (i). Oxidative/nitrative stress (DNA damage, lipid peroxidation, protein alteration, and therefore mutation) is caused by the production of ROS (ii) and NO (iii) by leukocytes. Oxidative stress is increased when antioxidant enzymes (iv) and antioxidants (v), which scavenge ROS, are reduced. Chronic inflammation requires a positive feedback loop between pro‐inflammatory cytokines (vii) and NF‐κB (vi). When it comes to inflammation‐related carcinogenesis, anti‐inflammatory cytokines (viii) are downregulated. Leukocytes are drawn to inflammatory areas by chemokines (ix). Along with ROS, NO, and pro‐inflammatory cytokines, COX‐2 (x) inhibits immunosurveillance and apoptosis while promoting angiogenesis and cell proliferation. DNA methylation is another effect of inflammation that leads to abnormal gene expression. The red boxes indicate ten potential chemopreventive targets. The green boxes indicate factors that have been reduced. T‐shaped arrows show suppression, while pointed arrows show activation or promotion [95]. Adapted from Kanda et al. (2017), International Journal of Molecular Sciences, published under CC BY 4.0 license.

**FIGURE 5:**
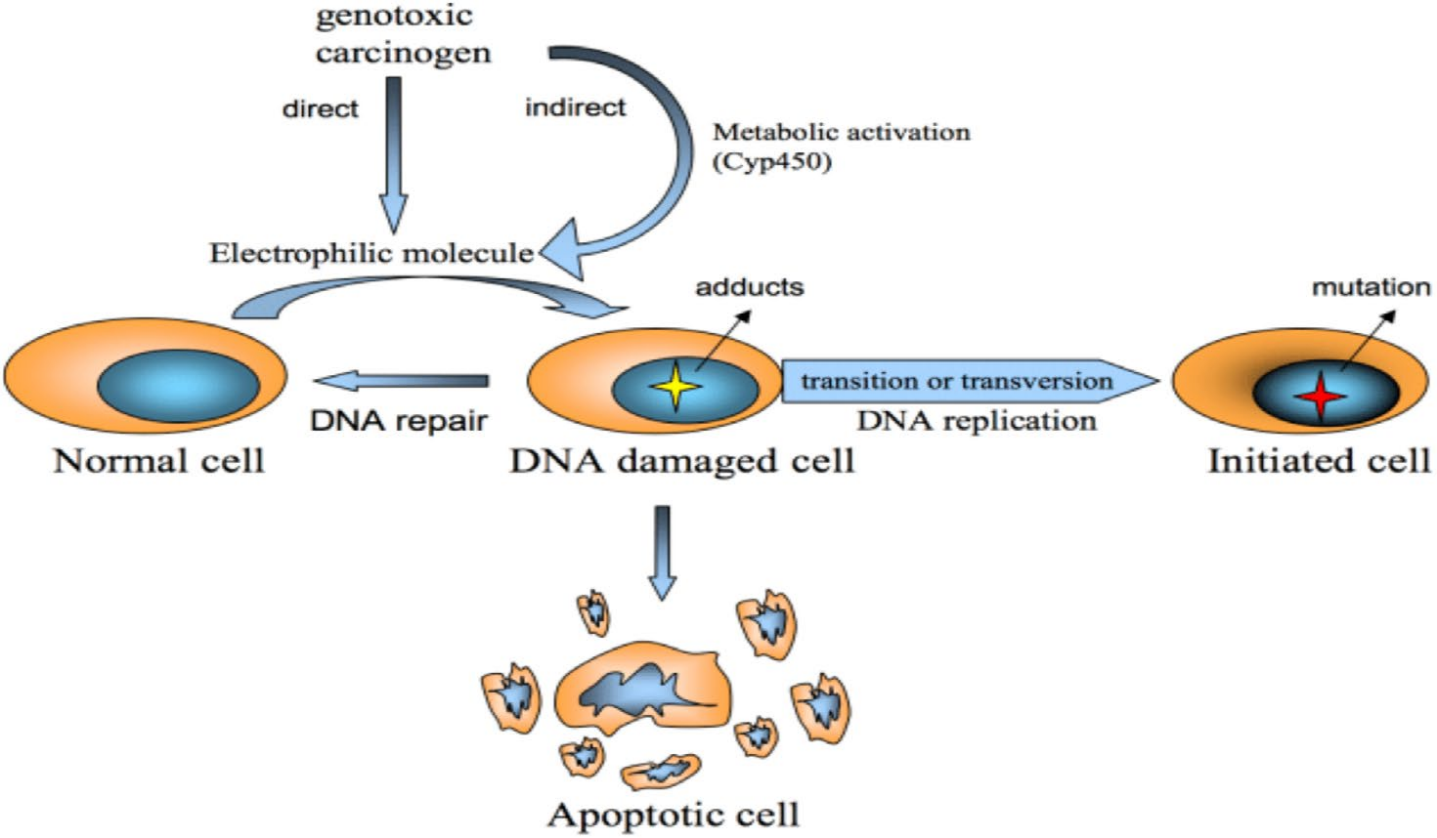
Molecular Mechanisms of Genotoxic Carcinogen. Chemical carcinogens may directly or indirectly (metabolic activation) be converted to electrophilic molecules that interact with DNA. Depending on the cell injury level (formation of DNA‐adducts), different ways of cellular response may occur; with low damage level, cells can be reverted to normal by DNA repair, and with excessive damage, cells can undergo programmed cell death; however, under cellular replicative pressure, damaged DNA allows cell mutation, thus initiated cells carry permanent and heritable DNA changes that may predispose to cancer development [96]. Adapted from Camacho (2012), with permission from Bentham Science Publishers.

There are several categories of drug‐induced carcinogenesis, which are described by a number of molecular pathways (Table 3; Figures 3, 4, 5) [93].

**TABLE 3:** Molecular mechanisms of drug‐induced carcinogenesis.

Mechanism	Description	Key drug examples	Cellular consequences	References
DNA damage and mutations	Direct interaction with DNA causing: DNA adducts; Strand breaks; Base modifications	Alkylating agents (cyclophosphamide); platinum agents (cisplatin); azathioprine	Gene mutations; chromosomal aberrations; activation of oncogenes; inactivation of tumor suppressors	[65, 66, 67, 68, 69, 70, 71, 72]
Epigenetic alterations	Heritable gene expression changes without DNA sequence alteration: DNA methylation; Histone modification; Chromatin remodeling	DNA methyltransferase inhibitors; HDAC inhibitors; arsenic trioxide	Silencing tumor suppressor genes; activation of oncogenes; genomic instability	[73, 74, 75, 76]
Immune suppression	Impaired recognition and elimination of: Virus‐infected cells; Premalignant cells; Malignant cells	Calcineurin inhibitors (cyclosporine); azathioprine; TNF‐α inhibitors	Viral oncogenesis (EBV, HPV, HHV‐8); accumulation of DNA‐damaged cells; escape from immune surveillance	[77, 78]
Receptor‐mediated signaling	Dysregulation of hormone signaling: Genomic (nuclear receptor) pathways; Non‐genomic (membrane receptor) pathways	Estrogen therapy; tamoxifen; androgen therapies	Sustained proliferation; resistance to apoptosis; ROS generation; chromosomal instability	[79, 80, 81, 82, 83, 84]
Chronic inflammation	Persistent inflammation creating pro‐carcinogenic microenvironment: Immune cell infiltration; Cytokine production; Oxidative stress	Multiple agents (indirect mechanism)	ROS/RNS → DNA damage; Cytokine‐mediated proliferation; extracellular matrix remodeling; angiogenesis	[85, 86, 87, 88, 89, 90, 91, 92]

### 4.1 DNA Damage and Mutations

Numerous chemotherapeutic and immunosuppressive drugs directly create adducts of DNA, resulting in strand breaks or disrupting the repair of potential DNA damages [65]. Examples of such an agent include alkylating (e.g., cyclophosphamide), platinum‐based (e.g., cisplatin) and azathioprine based drugs, which induce mutations in genes that control cell division, cell growth and cell apoptosis [66, 67]. These genetic mutations may either activate oncogenes (e.g., RAS) or silence tumor suppressor genes (e.g., TP53), triggering clonal growth and cancer invasion [68].

Most recently, the use of single‐cell multi‐omics has allowed scientists to define how events unfold in time starting with initial DNA damage to subsequent clonal selection and evasion by immune response of the consequences of patient‐derived models [69]. These methods have shown that mutations caused by drugs usually conform to certain patterns or “mutational signatures” that could be distinctly linked either to particular classes of drug [70, 71]. As an example, alkylating agents leave characteristic signatures that are characterized by C>T transitions at CpGs, whereas the platinum agents leave unique patterns of crosslink‐linked mutations. Knowledge about such signatures can support detection of pre‐malignant clones earlier before they progress to stage IV cancer as well as risk stratification [42, 72].

### 4.2 Epigenetic Alterations

Changes in the DNA that can change gene expression without involving a change in the DNA sequence: Epigenetic changes such as the methylation of DNA, histone modification, and chromatin remodeling can change gene expression [73]. A number of the drugs enhance carcinogenesis by stimulating the process of epigenetic changes which silenced tumor suppressor genes or activated oncogenes. As an example, arsenic trioxide depletes, and SAM causes global hypomethylation of DNA and genomic instability [74, 75]. New CRISPR‐based screening technology is being applied to determine which genes make an individual prone to drug‐induced epigenetic changes which could result in finding new targets to attack for preventing cancer [76].

### 4.3 Immune Suppression

Immunosuppressive drugs have the capacity to impair the capacity of the immunological system to identify and destroy cell infections and cancer. This dysfunctional state of immune surveillance gives the precancer cells an opportunity to survive, multiply, and eventually develop tumors. Two pathways are involved in carcinogenesis in immunosuppression [77, 78].

### 4.4 Receptor‐Mediated Signaling Pathways

Using hormonal therapies has oncogenic effects caused by dysregulation of receptor‐mediated signaling pathways which regulate cell proliferation and survival in a normal manner [79, 80]. An example of hormones is estrogen which increases cell growth and survival through the binding of estrogen receptors (ERa and ERb) which generate signal transduction cascades that modify gene transcription [81, 82]. In addition to the traditional genomic pathways, new information focuses on the relevance of ligand independent ER activation, wherein the growth factors (i.e., IGF‐1, EGF) activate and phosphorylate ERs even in low‐estrogen conditions, which results in self‐reinforcing proliferative loops [83, 84].

#### 4.4.1 Other Pro‐Carcinogenic Effects of Hormonal Signaling Are

- Development of reactive oxygen species (ROS) during estrogen metabolism, which results in oxidative genomic instability damage on DNA [85, 86].
- Aneuploidy and chromosomal instability as a result of blockage of mitogenic fidelity [87].
- Angiogenesis stimulation and anti‐apoptotic effects: Generation of a microenvironment conducive to tumor growth [88].

The combination of genetic, epigenetic, and microenvironmental damages changes physiological growth signals into sufficiently potent carcinogenic stimuli [89, 90].

1. Indirect Mechanisms (Loss of Immuno‐surveillance): This is the standard view in which the body's normal mechanisms for eliminating the following become blunted by the dampening effect on the immune system:

Virus‐infected cells (e.g., EBV → PTLD, HPV → cervical cancer, HHV‐8 → Kaposi's sarcoma, HCV → hepatocellular carcinoma) [90].

Premalignant and malignant cells damaged from DNA are generated daily and accumulate.

2 Direct Mechanisms (Oncogenic Pathway Activation): This is the new and much more underhanded one. The drugs would then be those that directly modulate intracellular signaling pathways controlling apoptosis, cell cycle progression, DNA repair, and angiogenesis. They then promote cancer's hallmark features [91, 92].

## 5 Risk Factors

Drug‐induced carcinogenesis can be dependent on various host‐related factors, candidate drugs, and environmental factors [97].

The vast majority of approved drugs do not induce cancer, although some, especially highly potent ones—how, chemotherapy agents—are deemed carcinogenic.

### 5.1 Drug Specific Factors

The chemical and biological qualities ascribed to the features of the drug have a bearing on its carcinogenic potential. The IARC has identified ten “key characteristics” representing potential mechanisms through which an agent might be capable of causing cancer [98].

- Electrophilicity and metabolic activation: Some types of carcinogens are electrophilic or electron‐seeking, reacting directly with intracellular structures such as DNA, proteins, and lipids to form adducts. Others, called “procarcinogens”, require metabolic activation in the body to create reactive electrophiles [98, 99].
- Genotoxicity: The capacity of a drug to directly damage a target cell's genetic material is associated with the onset of carcinogenicity. This may result in the formation of DNA adducts, strand breaks, and mutations, all of which initiate the carcinogenic process [100].
- Dose and duration: The dose and duration of drug exposure are critical parameters. Long‐term, high‐dose exposure to a non‐genotoxic drug that produces chronic toxicity and cell proliferation might increase the risk for carcinogenesis [101].
- Hormonal action: A drug that alters hormone levels may promote specific cancers. For example, hormone therapies for postmenopausal women have been linked with an increase in the risk of breast and endometrial cancer [102].
- Contamination: Carcinogenic agents may be introduced in the manufacture or during the storage of the drugs. A good example is that of diabetes drugs found to be contaminated with nitrosamines, known to be carcinogenic, and hence recalling them from the market [103, 104].
- Immunosuppression: Immunosuppressive agents are rather associated with an increased risk for developing cancer in the sense that they suppress the normal immune surveillance function—that generally eliminates any cancer cells [105].

### 5.2 Patient‐Specific Factors

The unique biological constitution and health status of the host could either contribute to or reduce susceptibility toward therapeutic agents' carcinogenic effects.

- Genetic predisposition: Pharmaceutical genetics is the study of how genetic variations influence the response to drugs by individuals. Genetic polymorphisms in drug‐metabolizing enzymes and transporters can modify the effectiveness and reach toxic levels when relating to carcinogenicity [106]. For example, polymorphism causing irrevocable alterations in certain drug metabolism within a patient may be attributable to variations in the cytochrome P450 enzyme (CYP) genes [107].
- Age: The risk for cancer inflicted by drugs is age‐dependent during exposure; generally, there is a long latency of many years to several decades between exposure time and eventual emergence of cancers [108]. People of older age are more susceptible to a higher prevalence of cancer due to the ageing process along with the damage to the immune system and the DNA repair mechanisms [109].
- Medical history: Any co‐morbid medical condition present in the patient such as immune deficiency, or perhaps another malignancy would have an entirely different risk profile for drug‐induced carcinogenicity [110].
- Lifestyle factors: Behavioral factors—alcohol behavior, smoking behavior, diet, and obesity in general—are all risk modifiers of the cancer risk of medications [111]. The combination of tobacco use and exposure to some drug or environmental carcinogens is most likely to yield multiplicative increases in risk [112].

### 5.3 Environmental Factors

External factors in a patient's environment and concurrent exposures can affect the risk of drug‐induced cancer.

- Drug–drug interactions: Taking multiple drugs simultaneously can lead to complex interactions that alter how each one is metabolized, possibly increasing the concentration of a toxic metabolite [113]. Some selective serotonin reuptake inhibitors (SSRIs), for example, would inhibit the metabolism of tamoxifen, reducing its effectiveness [114].
- Concurrent carcinogen exposure: Exposures to environmental carcinogens such as asbestos, benzene, or radiation may have synergistic effects with drugs so as to enhance the risk of cancer [112, 115].
- Viruses and bacteria: Some infective agents are known carcinogens, like human papillomavirus (HPV) or *Helicobacter pylori* , and can interfere with drug therapies [116]. For example, the use of immunosuppressive medication can impair immune surveillance and facilitate the reactivation of latent oncogenic pathogens, making an individual more vulnerable to cancer caused by viruses [117].

## 6 Clinical Recommendations and Decision‐Making Frameworks

The risk of carcinogenicity associated with certain drugs necessitates a forward‐oriented and structured clinical approach to risk assessment and mitigation. The framework below summarizes recent evidence‐based recommendations to assist clinicians in their decision‐making prior to and while using a potentially carcinogenic drug [118].

### 6.1 Pre‐Therapy Risk Assessment and Stratification

- Medical history: A detailed past personal and family medical history of cancer, including cancer type and age at onset. Assessment of modifiable risk factors (e.g., smoking, alcohol, and exposure to UV light) and history of oncogenic viral infections (e.g., HPV, HBV, HCV, EBV) is important measure [119].
- Pharmacogenomic testing: In cases of available technology implement pre‐emptive pharmacogenomic testing for genetic variations affecting drug metabolism and subsequent cancer risk. For example: (1) Testing for variants of thiopurine S‐methyltransferase (TPMT) or nudix hydrolase 15 (NUDT15) is recommended before commencing thiopurine (azathioprine) therapy to determine patients at high risk for drug toxicity and secondary malignancies related to toxicity. (2) Pre‐testing for variants of dihydropyrimidine dehydrogenase (DPYD) is indicated prior to treatment with fluoropyrimidine chemotherapy to prevent the risk of severe toxicity and subsequent complications [120, 121].
- Baseline cancer screening: Prior to starting any long term high risk therapy (e.g., dermatologic examination for patient candidate to start long term immunosuppressants, and skin cancer, gynecology examination with pelvic ultrasound for women starting tamoxifen, CBC monitoring for patients considered to be treated with alkylating agents), dependent on age and stratified risk, baseline and annual screening for cancer should be completed [30, 56].

### 6.2 Drug and Regimen Selection

- Principle of safer alternatives: Whenever there are therapeutically equivalent options, prior selection should be agents with less known carcinogenic potential. For example, in the case of immunosuppressants, the use of MMF may be preferred instead of azathioprine where possible, as part of CNI‐sparing regimens [59].
- Informed consent (Risk–benefit discussion with patients): Engage patients in the process of shared decision‐making about their medications. Be clear to patients about the absolute versus relative risk of their cancer in comparison to the established benefits of the drug for their condition (e.g., cyclophosphamide has a survival benefit in vasculitis; this benefit needs to be carefully weighed against the risk of leukemia) [122].

### 6.3 Ongoing Monitoring and Surveillance

- Risk‐adopted surveillance plans: Create an individual monitoring schedule based on the drug risk profile and patient‐specific risk factors.
- Immunosuppressants: Every year for skin cancer, have an annual dermatologic examination and evaluation for lymphadenopathy [56].
  - Tamoxifen: Annual gynecologic assessment and prompt investigation of abnormal uterine bleeding [123].
  - Alkylating agents/topoisomerase inhibitors: Routine complete blood counts for myelodysplastic syndrome (MDS) and acute myeloid leukemia (AML) for years following cessation of treatment
- Lifestyle modification: Risk reduction of the patients should be given particular advice, which includes:
  - Protective measures against sun rays in immunosuppressed patients [56].
  - Smoking cessation support [119].
  - Loss of weight and nutritious diet [119].

### 6.4 Treatment Mitigation Strategies

Mitigation strategy involves integration of both conventional and a new and applicable novel. The novel approach produces a more active, technologically advanced, and patient‐focused approach to managing medication risks, including those that may result in cancer [124].

#### 6.4.1 Controlled Writing of Prescriptions: AI‐Driven Risk Assessment

##### 6.4.1.1 Conventional Method

Providers carry out a manual risk–benefit analysis based on the patient's medical history and comorbid risk factors [125].

##### 6.4.1.2 Combined Novel Method

- AI‐enhanced risk–benefit analysis: Medication prescribing using AI and advanced machine learning tools helps evaluate the patient's digital health profile, together with anonymized electronic health records, genetic markers, environmental factors, lifestyle data, and others [124, 126].
- Predictive risk scoring: The AI tool calculates the cancer risk from the medication. It uses complicated patterns that humans fail to capture, especially with several risk factors, and provides an advanced risk–benefit analysis to clinicians [126].
- Pharmacogenomic guidance: AI's recommendations can be complemented by pre‐emptive pharmacogenomic testing to enable prescribers to pick the most appropriate medication and dose for the patient based on their genetic profile [127, 128].

#### 6.4.2 Dose Optimization: Real‐Time Therapeutic Monitoring

##### 6.4.2.1 Conventional Approach

The minimum effective dose is mandated to reduce the risk of cancer [129]. The dose modifications are based on infrequent visits and an unchanging view of the patient's health.

- Chemoprevention: Adjunctive agents may be considered for high‐risk populations. For example, topical diclofenac and oral nicotinamide have demonstrated effective in reducing in high‐risk populations with subsequent non‐melanoma skin cancers (NMSCs), immunocompromised patients [130].
- Vaccination: Where applicable, administration of HPV and HBV vaccination should be conducted prior to initiation of immunosuppression therapy [131].

##### 6.4.2.2 New Integrated Approach

- Continuous wearable monitoring: Patients use smart devices like watches and biosensors, which smartly measure biometrics, biomarker concentrations, and other physiological parameters [132].
- Data‐informed dose modification: Safely transmitting and analyzing data from these devices in real‐time through data‐driven dose adjustment [133]. If the patient's response indicates that a lower dose is required or if early adverse effects are observed, the doctor sends an alert. Rather than making static adjustments, the dose can be dynamically optimized in real‐time. This is advantageous [134, 135].
- Therapeutic window management: This method ensures that the drug remains in an appropriate dosage range at all times while maintaining efficacy [136, 137, 138].

#### 6.4.3 Detection and Monitoring: Predictive Surveillance Using Blockchain Technology

In order to detect cancer early, patients who are taking potentially harmful drugs undergo regular checks and monitoring.

##### 6.4.3.1 Traditional Approach

While patients on certain medications with possible carcinogenic properties are accompanied by regular check‐ups and surveillance aimed at the early stages of cancer, which is rather more passive than active [139].

##### 6.4.3.2 Integrated Novel Approach

- Predictive, AI‐powered surveillance: Using AI based risk profiling for refinement of the surveillance, the system can actively flag and schedule for enhanced targeted surveillance on these patients and automatically prioritize examinations [140].
- Decentralized, secure patient health records (Blockchain): Patient monitoring, including data on the treatment and the outcome, progressive imaging, and biomarker evolution, is stored on a patient‐centered secure, distributed blockchain system [141, 142].
- Transparent and interoperable records: Such systems are designed to maintain non–disputable and non–editable patient records for automatic data collection, for epidemiological reasons, and longitudinal surveillance on the patient for authorized entities. They propagate the system's proof of active visibility during the longitudinal surveillance and the auditable mechanisms for research [143, 144].

#### 6.4.4 Alternative Therapies: The Role of AI in New Therapy Discovery

##### 6.4.4.1 Traditional Approach

Physicians target less risky alternative therapies as part of their plan [145]. This is mostly constrained by a physician's knowledge and the available clinical frameworks.

##### 6.4.4.2 Integrated Novel Approach

- Expanded search with AI: PhD graduates and other professionals in the fields of Artificial Intelligence and Machine Learning are capable of using these tools to search through databases of pharmaceutical information, medical literature, and patient outcomes so as to identify and assess alternative therapies that may be safer than the current therapies, which may include repurposed and experimental drugs (Table 4) [150].
- Personalized alternative recommendations: The AI in the physician's therapy recommendation can identify other therapies that can substitute a particular therapy, taking into account the patient's unique health conditions and genetic profile, as opposed to the typical standardized approach [151, 152].
- Trial integration: In case of new therapies, the system can assist in integrating patients into relevant clinical trials, with blockchain technology potentially governing the secure management of research data [146].

**TABLE 4:** Risk factors and mitigation strategies for drug‐induced carcinogenesis.

Category	Risk factor	Description and impact	Current evidence‐based strategies	Emerging approaches*	Citations
Drug factors	Genotoxicity	Direct DNA damage capacity: DNA adduct formation; Strand breaks; Chromosomal aberrations	Preclinical genotoxicity testing (regulatory requirement); Avoid combinations with genotoxic potential	Machine learning prediction models; In silico screening tools (early development)	[98, 99, 100]
	Dose and duration	Cumulative exposure increases risk: Linear dose–response for genotoxic agents; threshold effects for non‐genotoxic drugs	Minimum effective dose; regular review of continued need; Treatment breaks when clinically feasible	Real‐time therapeutic drug monitoring (limited data); Wearable biosensors (research setting only)	[101, 127, 130, 131, 132, 133]
	Immunosuppression	Impaired immune surveillance: Viral oncogenesis (EBV, HPV, HHV‐8); Escape of DNA‐damaged cells	Lowest effective immunosuppression; CNI‐sparing regimens; mTOR inhibitors as alternatives	Immune function biomarkers (investigational); T‐cell repertoire monitoring (research use)	[59, 105, 138]
Patient factors	Genetic predisposition	Polymorphisms in drug‐metabolizing enzymes: TPMT, NUDT15; DPYD; CYP450 variants	Pre‐emptive pharmacogenomic testing: TPMT/NUDT15 before thiopurines; DPYD before fluoropyrimidines; dose adjustment based on genotype	Polygenic risk scores (under validation); Integration with electronic health records (in development)	[106, 107, 120, 121, 124, 126]
	Age	Older adults: accumulated damage; Children: longer latency, developing tissues	Age‐adjusted risk assessment; enhanced surveillance in elderly; consider pediatric latency periods	Biological age markers (research); DNA repair capacity assays (preclinical)	[108, 109, 124]
	Lifestyle factors	Smoking, alcohol, obesity, UV exposure (synergistic effects with medications)	Counseling on modifiable risks; Smoking cessation; Sun protection; Weight management support	Digital health interventions (under study); Personalized risk feedback (prototype stage)	[111, 112, 119, 146, 147, 148]
External factors	Drug–drug interactions	Altered metabolism leading to toxic accumulation: CYP450 inhibition/induction; Transporter competition; additive toxicity	Comprehensive medication reconciliation; avoid known interactions; consult drug interaction databases	AI‐powered prescribing systems with interaction alerts (limited validations)	[113, 114, 124, 125, 126, 144]
	Viral/bacterial co‐infections	Oncogenic pathogens: HPV, HBV, HCV, EBV, HHV‐8; *H. pylori*	Pre‐treatment screening: HPV, HBV, HCV, EBV serologies; vaccination before immunosuppression: HPV, HBV vaccines; Antiviral prophylaxis when indicated	Therapeutic vaccines (investigational); CRISPR‐based antiviral therapies (preclinical)	[116, 117, 119, 129, 149]

#### 6.4.5 Patient Education: Patient Controlled Digital Pharmacovigilance

##### 6.4.5.1 Traditional Approach

Patients are educated on these risks and are explained the importance of monitoring their health [147, 153]. Patients are the main stakeholders in the compliance process.

##### 6.4.5.2 Integrated Novel Approach

- Interactive Mobile Health apps: Patients are no longer confined to brochures; they now have access to health applications that provide tailored and AI‐generated texts on their medications, associated risks, and monitoring plans [154].
- Real‐time adherence monitoring: The app is capable of monitoring patient adherence to medication using ‘smart pill bottles’ and other internet‐enabled devices and sending tailored reminders (Table 4) [149].
- Patient control via blockchain‐empowered technology: An app integrated alongside blockchain technology enables patients to have full concentration over their personal medical information. This encouragement and empowerment allows patients to determine who can view their data, which enhances their engagement toward treatment compliance [146, 148].
- Gamified engagement: Adherence and monitoring tasks can be accomplished through ‘gamification’ features that have been designed to reduce the burden placed on patients and increase entertainment value [155, 156].

## 7 Future Directions in Research on Drug‐Induced Carcinogenesis

The knowledge of carcinogenesis with drugs is a potential source of research. The future stratum of work must shift its paradigm of observational studies to a mechanistic comprehension, predictive instruments, and preventive interventions. The following techniques are still at preliminary research and would have to be confirmed by future clinical trials before they can be utilized in a clinical setting.

### 7.1 Emerging Technologies Under Investigation

#### 7.1.1 AI in Risk Assessment

Machine learning applications are under development in order to combine patient data (electronic health records, genetic markers, environmental factors, lifestyle data) to predict personalized risk of cancer. One set of studies indicates that such tools could specify the complex risk patterns that cannot be easily discerned by clinicians, although future studies should validate them [124, 126].

#### 7.1.2 Wearable Devices Monitoring

The wearable biosensors may be used in continuous monitoring to measure biometric parameters and physiological changes during drug therapy [132, 133]. Dynamic dose adjustment in real‐time could be made possible by data transmission eventually, but proof‐of‐concept investigations can be seen at present [134, 135].

#### 7.1.3 Blockchain for Health Records

The distributed ledger technology is also being considered to provide secure, patient‐centered health records storage [141, 142]. Its possible usages are clear observation of longitudinal surveillance data and research audit documents, but considerable technical and regulatory obstacles still persist [143, 144].

### 7.2 Mechanistic Research and Biomarker Development

- Specific aim: Move from correlative data to causal understanding of how certain classes of drugs (e.g., immunosuppressants, hormone therapies, chemotherapies) initiate and promote tumorigenesis [157, 158]
  - Single‐cell multi‐omics: Researchers are increasingly able to construct a time course of patient‐derived models of initial DNA damage to clonal selection using the more sophisticated genomic methods [129]. These methods have demonstrated drug‐specific mutational signatures which could be more useful in detecting pre‐clonal clones early [130, 131].
  - Liquid biopsy in early detection: Field carcinogenesis Liquid biopsy assays that identify drug‐specific mutational signatures or abnormal patterns of methylation in cell‐free DNA are in research to understand how it is first identified [159, 160]. Cell‐free mitochondrial DNA can also be used as a possible sentinel biomarker of cellular stress induced by drugs [161, 162].
  - CRISPR‐based screening: Genome‐wide CRISPR‐Cas9 knockout or activation screens in human cell lines are now in use to determine genes that increase or decrease vulnerability or resistance to drug‐induced carcinogenesis [137, 138]. This could uncover new synthetic lethal interactions and targets to mitigate the outcomes.

### 7.3 Pharmacogenomics and Risk Stratification: Towards Personalized Prescribing

- Specific aim: Genetically actionable biomarkers will be developed to predict the inherent risk of developing cancer from a medication [163].
- Actionable research proposals:
  - Polygenic risk scores (PRS): Either the data from major biobanks (e.g., UK Biobank, All of Us) will be used to produce PRS composed of variations in genes involved in drug metabolizing enzymes (e.g., CYP450 family), DNA repair pathways (e.g., BRCA1/2, MLH1/MSH2), and immune function genes. These scores would be validated prospectively to inform drug selection [164, 165].
    Emerging understanding: Recent advances in pharmacogenomics, like Polygenic Risk Scores (PRS), are helping researchers determine the underlying risk for an individual to develop cancer from a drug. PRS are based on large biobanks and can shed light on how to use the future drug choice prospectively based on variations in genes associated with drug metabolism pathways and DNA repair pathways [166].
- Pre‐emptive genotyping trials: Substances Clinical trials to test whether pharmacogenomic stratification (Drug A vs. Drug B based on patient genotype) would decrease pre‐malignant biomarkers or incidence of cancer during long‐term follow‐up are currently undergoing trials [167].
- Key question: Can a specific haplotype in the TPMT or DPYD gene predict not only toxicity but also long‐term cancer risk in patients treated with thiopurines or fluoropyrimidines? [168, 169]

### 7.4 Focused on Safe Drug Development

- Specific aim: Integrate the risk assessment on carcinogenicity early in the drug design process to produce “safer‐by‐design” therapeutics [170].
- Actionable research proposals:
  - In silico prediction: The machine learning models that are being developed have integrated chemical structure information, −omics information, and high‐throughput screening information to predict the pro‐carcinogenicity in early drug design [171]. The strategies focus on facilitating safer‐by‐design therapeutics.
  - Prodrug strategies for tissue targeting: Prodrug designs that target tissues (e.g., tumors) are also under investigation in order to minimize the general exposure of the system and off‐target DNA damage in healthy organ [172].
  - Key question: Can we, indeed, substitute the metabolically activated moiety in Drug Y with a novel chemical group that would retain therapeutic efficacy but cannot be metabolized into a DNA‐alkylating agent [173]?

### 7.5 Novel Mitigation Strategies

- Senolytic therapies: Preclinical trials are looking into the possibility that senolytic agents may be temporarily added to the treatment breaks to remove senescent cells generated by drug‐induced damage and decrease the extent of cancer that may follow [174, 175].
- Gene editing strategies: Base editing and prime editing of HSCs are under development to remodel patients who need chronic P53‐mediated high‐risk immunosuppression with protective mutations (e.g., TP53 hyperactive) [176, 177]. These methods are experimental and there are many safety and ethical aspects that have to be considered.

## 8 Global Perspectives on Prescription Trends and Disparities

- Specific aim: Understand and address global inequalities in exposure to procarcinogenic drugs and the associated disparities in cancer risk [178].
- Actionable research proposals:
  - Pharmacoepidemiology in diverse population: Conduct a multinational pharmacoepidemiological comparison of high versus low incidences of specific cancers in populations that vary by generic utilization (e.g., proton pump inhibitors, selected chemotherapies) in low/middle‐income countries (LMIC) versus those in high‐income countries (HIC) [179].
  - Digital clinical decision support (Interventional Studies): Implement and assess a digital clinical decision support designed primarily for use in LMIC primary care settings for flagging high‐risk prescribing patterns (e.g., for long‐term proton pump inhibitor use without indication) to suggest safer alternatives [180].
  - Research on health disparities: It requires studies to be conducted regarding the possible variations in the hormone‐related cancer risks in the context of long‐term use of contraceptives in relation to geographic areas, genetic ancestry, and access to formulations [181, 182].
  - Key question: Are there differences in the risk for hormone‐related cancers associated with long‐term contraceptive use by geographic region, genetic ancestry, or access to alternative formulations, and what policies can address this [183, 184, 185]?

## 9 Conclusion

Carcinogenicity of selected essential medicines has become a major problem in modern therapy. The time for change is now, and it must be radical and transformative going from passive surveillance to proactive, predictive risk management. Integration of artificial intelligence into the personalized risk assessment service, pharmacogenomics in stratified prescribing, and blockchain‐secured real‐time monitoring will usher in a new era of precision medicine. This technological advantage will allow us to minimize, if not completely eliminate, the iatrogenic risks while maintaining therapeutic benefit. Eventually, turning this understanding into clinical action becomes necessary to secure patients and meet the primary mandate of medicine: premium non nocere—first, do no harm.

## Author Contributions

**Awgichew Shewasinad Yehualashet:** writing – review and editing. **Kassahun Dires Ayenew:** conceptualization, writing – review and editing, supervision. **Awol Mekonnen Ali:** writing – review and editing. **Kalkidan Tekletsadik:** writing – original draft. Desta Seyoum Tadesse and Kalkidan Tekletsadik have conducted the review. Berhan Begashaw, Awol Mekonnen Ali and Awgichew Shewasinad Yehualashet provided editorial corrections. Kassahun Dires Ayenew has supervised the review. All authors have approved the final version.

## Funding

The authors have nothing to report.

## Ethics Statement

The authors have nothing to report.

## Consent

The authors have nothing to report.

## Conflicts of Interest

The authors declare no conflicts of interest.

## Data Availability

The data that support the findings of this study are available on request from the corresponding author. The data are not publicly available due to privacy or ethical restrictions.
